# Amperometric Sensor for Detection of Chloride Ions^†^

**DOI:** 10.3390/s8095619

**Published:** 2008-09-15

**Authors:** Libuse Trnkova, Vojtech Adam, Jaromir Hubalek, Petr Babula, Rene Kizek

**Affiliations:** 1 Department of Chemistry, Faculty of Science, Masaryk University, Kotlarska 2, CZ-611 37 Brno, Czech Republic; 2 Department of Chemistry and Biochemistry, Faculty of Agronomy, Mendel University of Agriculture and Forestry, Zemedelska 1, CZ-613 00 Brno, Czech Republic; 3 Department of Animal Nutrition and Forage Production, Faculty of Agronomy, Mendel University of Agriculture and Forestry, Zemedelska 1, CZ-613 00 Brno, Czech Republic; 4 Department of Microelectronics, Faculty of Electrical Engineering and Communication, Brno University of Technology, Udolni 53, CZ-602 00 Brno, Czech Republic; 5 Department of Natural Drugs, Faculty of Pharmacy, University of Veterinary and Pharmaceutical Sciences, Palackeho 1-3, CZ-612 42 Brno, Czech Republic

**Keywords:** Chloride Ions, Silver, Carbon Paste Electrode, Screen Printed Electrode, Voltammetry, Amperometry

## Abstract

Chloride ion sensing is important in many fields such as clinical diagnosis, environmental monitoring and industrial applications. We have measured chloride ions at a carbon paste electrode (CPE) and at a CPE modified with solid AgNO_3_, a solution of AgNO_3_ and/or solid silver particles. Detection limits (3 S/N) for chloride ions were 100 μM, 100 μM and 10 μM for solid AgNO_3_, solution of AgNO_3_ and/or solid silver particles, respectively. The CPE modified with silver particles is the most sensitive to the presence chloride ions. After that we approached to the miniaturization of the whole electrochemical instrument. Measurements were carried out on miniaturized instrument consisting of a potentiostat with dimensions 35 × 166 × 125 mm, screen printed electrodes, a peristaltic pump and a PC with control software. Under the most suitable experimental conditions (Britton-Robinson buffer, pH 1.8 and working electrode potential 550 mV) we estimated the limit of detection (3 S/N) as 500 nM.

## 1. Introduction

Chloride ions sensing is important in many fields such as clinical diagnosis [1, 2] environmental monitoring [3-5] and various industrial applications [6, 7]. Considering the fact that chloride channels play crucial role in physiological processes it is not surprising that missregulation of chloride ions transport by these channels can cause serious disorders. Cystic fibrosis is a disease in which gene encoding of a protein, called cystic fibrosis transmembrane regulator, and which functions as a chloride channel in epithelial membranes, is mutated and thus its function is altered [8]. Besides the importance of monitoring of chloride ions in patients with this disease, monitoring of chloride ions in the environment is needed. Chloride ions content in concrete plays important role in the quality of reinforced concrete, as these ions induce depassivation of the steel rebars and initiation of the corrosion process leading to degradation of the structure. Chloride ions in concrete come from cement, aggregate materials and water used for creating concrete, or by diffusion of chloride ions from outside of the structure through water pores in the concrete. Determination of chloride ions in materials for concrete is thus necessary [3, 9]. The content of chloride ions in waters is also monitored well [10, 11].

Numerous analytical methods for chloride ions in a variety of samples have been developed, such as ion chromatography [17, 18] near-infrared spectrometry [12] spectroscopy [13] light scattering [14] ion-selective electrode method [6, 9, 15, 16] turbidimetric method [17] and flow based methods coupled with different detectors [9, 18, 19]. On the other hand, sensors and biosensors have the advantages of specificity, low cost, ease to use, portability and the ability to furnish continuous real time signals [20-66]. Several sensors or biosensors for selective chloride ions detection have been suggested [1, 3, 6, 10, 15, 67-71]. The main aim of this paper was to test various carbon paste electrodes for detection of chloride ions. Moreover, we adopted the results obtained to suggest miniaturized device for *in situ* monitoring of chloride ions.

## 2. Material and Methods

### 2.1 Chemicals, material and pH measurements

Chemicals used were purchased from Sigma Aldrich Chemical Corp. (USA) in ACS purity unless noted otherwise. The stock standard solutions was prepared with ACS water (Sigma-Aldrich, USA) and stored in the dark at -4 °C. Working standard solutions were prepared daily by dilution of the stock solutions. The pH value was measured using WTW inoLab Level 3 with terminal Level 3 (Weilheim, Germany), controlled by the personal computer program (MultiLab Pilot; Weilheim, Germany). The pH-electrode (SenTix-H, pH 0–14/3M KCl) was regularly calibrated by set of WTW buffers (Weilheim, Germany).Deionised water underwent demineralization by reverse osmosis using the instruments Aqua Osmotic 02 (Aqua Osmotic, Tisnov, Czech Republic) and then it was subsequently purified using Millipore RG (Millipore Corp., USA, 18 M′Ω) – MiliQ water.

### 2.2 Voltammetric measurements

Voltammetric measurements were performed with an AUTOLAB Analyzer (EcoChemie, Netherlands) connected to a VA-Stand 663 (Metrohm, Switzerland), using a standard cell with three electrodes. The working electrode was a carbon paste electrode. The reference electrode was the Ag/AgCl/3 M KCl electrode, and a platinum wire was used as the auxiliary electrode. All experiments were carried out at room temperature. For smoothing and moving average baseline correction GPES 4.9 supplied by EcoChemie was employed. Experimental parameters of adsorptive transfer stripping technique (AdTS) coupled with differential pulse voltammetry (DPV) were as follows: step potential 5 mV, amplitude 25 mV, initial potential 0.05 V, end potential 1.1 V (more details are given in the Results and Discussion section). Britton-Robinson buffer consisted of 1:1:1 0.04 M boric acid (H_3_BO_3_), phosphoric acid (H_3_PO_4_) and acetic acid (CH_3_COOH) adjusted with 0.2 M NaOH to desired pH.

### 2.3 Preparation of carbon paste electrode and its modification

The carbon paste electrode (CPE) was made of 70 % (140 mg, *w*/*w*) graphite powder (Sigma-Aldrich) and 30 % (70 mg, *w*/*w*) mineral oil (Sigma-Aldrich; free of DNase, RNase, and protease). This carbon paste was housed in a Teflon body with a 2.5-mm diameter disk surface. Before measurements the electrode surface was renewed by wiping with wet filter paper. Then, the surface was ready for measurement of a 5 μL sample volume [72-75]. The silver modified-CPE was prepared in the same way as described above, with the exception of adding solution of AgNO_3_, solid form of AgNO_3_ or Ag particles (units of μm) into graphite powder and mineral oil. This mixture was ground to perfection for 10 min using agate grinding mortar.

### 2.4 Amperometric measurements

Amperometric measurements were carried out with multi-mode potentiostat BioStat (ESA, Inc. USA). It is four-channel system with three operating modes per channel (amps, volts, and temp). The system was connected through data bus USB to personal computer. The home made apparatus was connected to the first channel of the potentiostat. This apparatus consists of a basic plate on which the connector TX721 1115 with 2.54 pin spacing and the 0039532035 connector (Molex) with 1.25 mm pin spacing are placed. The connectors are designed for connection of two different screen-printed electrodes. Miniaturised electrode system consisted of working (silver), auxiliary (carbon) and reference (Ag/AgCl) electrode. Screen-printed electrodes are fabricated using standard thick-film techniques [76] on an alumina substrate forming the three-electrodes electrochemical sensor with dimensions of 25.4 × 7.2 mm [27]. Thick-film paste used for leads and contacts was AgPdPt based paste type ESL 9562-G (ESL Electroscience, UK). For auxiliary electrode Pt based paste type ESL 5545 was used. The working electrode was fabricated from Ag based paste type ESL 9912-K. Finally the reference electrode was fabricated from DuPont (DuPont, USA) paste type 5874 (Ag:AgCl ratio: 65:35).

### 2.5 Descriptive statistics

Data were processed using MICROSOFT EXCEL® (USA). Results are expressed as mean ± S.D. unless noted otherwise. The detection limits (3 S/N) were calculated according to Long and Winefordner [77] whereas N was expressed as standard deviation of noise determined in the signal domain unless stated otherwise.

## 3. Results and Discussion

### 3.1 Carbon paste electrode modified by solid AgNO_3_

**Figure 1.**
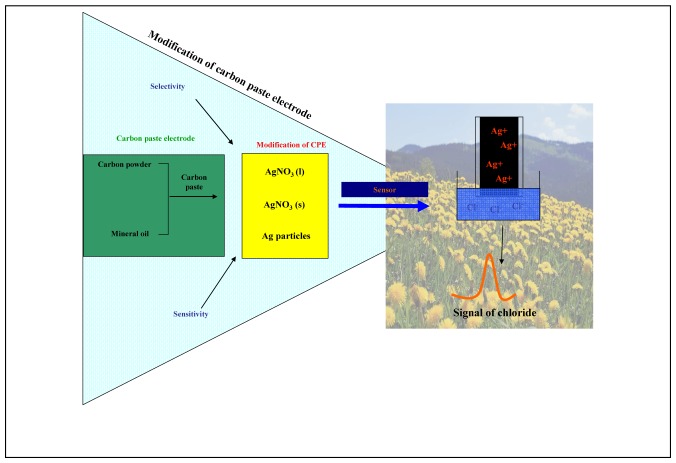
Chloride ion sensor Sensor is based on modification of a carbon paste electrode with silver (I). Reaction between silver (I) and chloride ions provides high selectivity and sensitivity for detection of chloride.

We measured chloride ions at the carbon paste electrode. The signal obtained was not well developed. The detection limit (3 S/N) was estimated down to 10^-4^ M. Therefore it was necessary to modify a working electrode (Figure 1). It is a common knowledge that silver (I) ions form a non-soluble AgCl precipitate with chloride ions. We used this feature to select suitable CPE modifiers to detect chloride ions. Primarily we choose solid silver (I) nitrate as the modifier (6 mg of AgNO_3_ was added to CPE).

**Figure 2.**
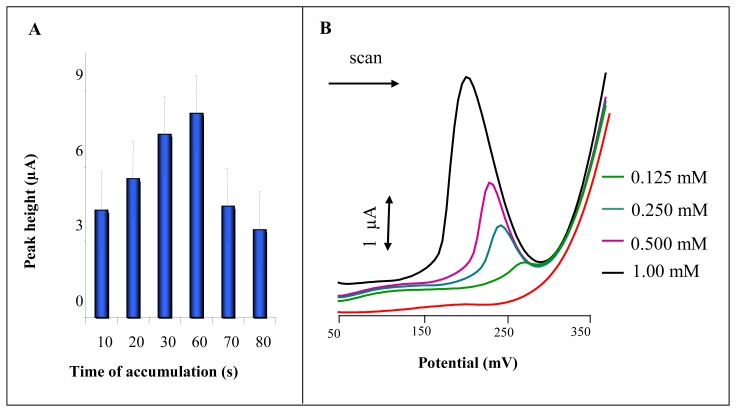
Electrochemical analysis of chloride ions using a CPE modified with solid silver(I) nitrate. (**A**) Dependence of signal height (100 μM NaCl) on accumulation time. Accumulation potential 250 mV. (**B**) DP voltammograms of NaCl (0.125, 0.250, 0.500 and 1.00 mM).

The CPE modified by solid silver (I) nitrate didn't give a signal in the presence of Britton-Robinson buffer (pH 1.8) only (not shown). Signal detected using modified CPE probably relates to formation of AgCl and its dissociation. Chlorides can be also adsorbed on the mercury electrode surface at +100 mV under formation of products with mercury similarly to silver electrodes surface. It was generally considered that this bond is too strong to be pure sorption. Their common superficial concentration is about 1.5 nmol/m^2^ [11, 78]. Therefore we tested the influence of accumulation of AgCl on the surface of modified CPE. Differential pulse voltammetric analysis was initiated by the accumulation of reaction product at potential +250 mV. The quantity of accumulated product increased with the time of accumulation up to 60 s, and then the signal declined (Figure 2A). The dependence of peak height on NaCl concentration within the range from 125 μM to 1 mM was studied (Figure 2B). The obtained signals were well developed and decreased from 1.2 μA to 0.2 μA with decreasing concentration of chloride ions. Potential of peak shifted to more positive potentials from 218 to 290 mV. Limit of detection (3 S/N) was estimated as 100 μM NaCl.

### 3.2 Carbon paste electrode modified by solution of AgNO_3_

In the following experiments the CPE was modified by aqueous silver(I) nitrate solution (0.4 μL of solution of AgNO_3_ 1 g/mL was added to the CPE). The CPE modified like this didn't give a signal in the presence of Britton-Robinson buffer (pH 1.8) only. After addition of 1 mM NaCl into the supporting electrolyte, a peak at 225 mV was determined. We observed that NaCl concentrations higher than 1 mM resulted in inhomogeneous saturation of modified CPE electrode surface by AgCl precipitate. This phenomenon caused nonlinearity of calibration curve in the range of the higher NaCl concentrations. The dependence of peak height on NaCl concentration within the range from 125 μM to 1 mM was linear, which shows on optimal ratio of aqueous silver (I) nitrate solution into CPE and concentration of chloride ions in supporting electrolyte. Therefore it can be also assumed that layer of AgCl precipitate on the surface of modified CPE is more homogenous. With increasing NaCl concentration potential of peak shifted to more positive potentials from 230 mV to 290 mV. Limit of detection (3 S/N) was estimated as 100 μM NaCl.

### 3.3 Carbon paste electrode modified by silver microparticles

**Figure 3.**
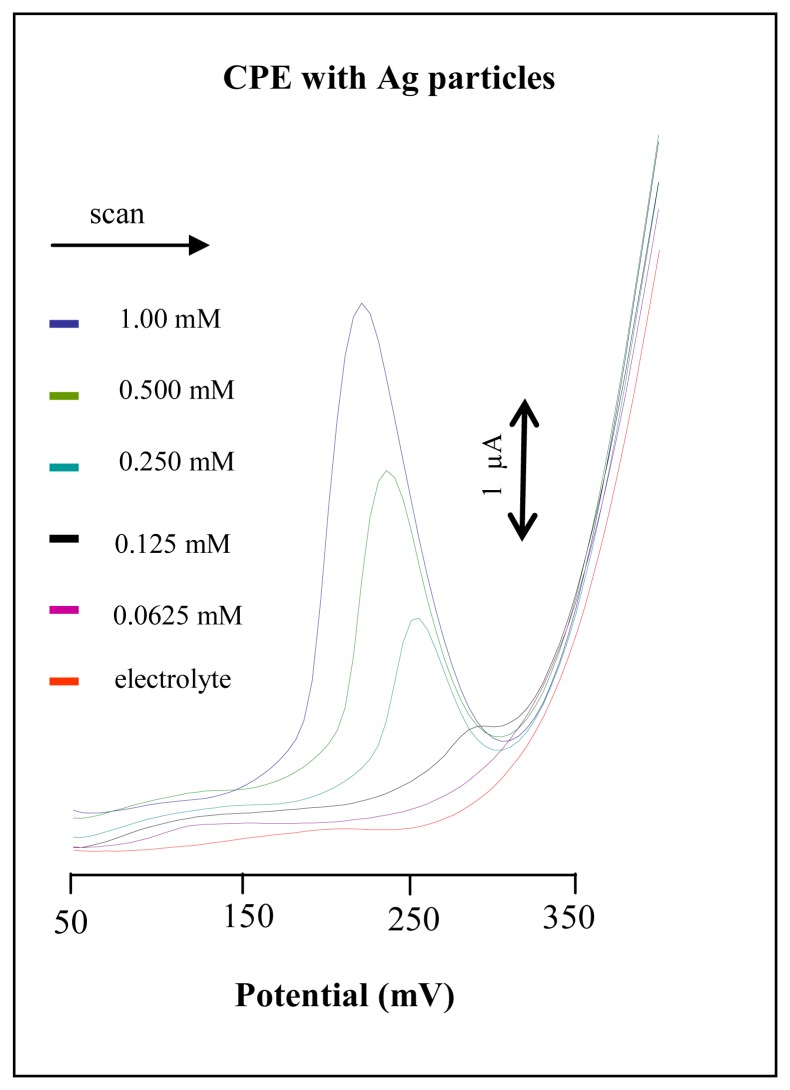
Electrochemical analysis of chloride ions using CPE modified by silver microparticles. DP voltammograms of NaCl (0.0625, 0.125, 0.250, 0.500 and 1.00 mM).

The CPE modified with silver microparticles (units of μm) is the most similar to a silver electrode. The additions of silver microparticles from 0.4 mg to 200 mg into carbon powder were tested. Silver microparticles additions below 50 mg didn’t provide sufficient sensitivity for chloride ion detection, because the detection limit (3 S/N) was higher than 250 μM NaCl. Silver microparticles contents higher than 160 mg made the CPE physical conditions worse; particularly a shorter shelf life of the working electrode and lower sensitivity were observed. We determined that the optimal composition of the modified CPE was 140 mg of Ag particles per 140 mg carbon powder (1:1, *w*/*w*). This rate provided sufficient sensitivity and shelf life longer than four weeks. Chloride ions were measured within the concentration range from 100 μM to 1 mM NaCl. DP voltammetric peaks were well developed with relative standard deviation below 5 % (Figure 3). Peak height decreased proportionally (from 8 μA to 0.2 μA) with decreasing chloride concentration and its potential shifted to more positive potentials (from 230 mV to 290 mV). Limit of detection (3 S/N) was estimated as 10 μM NaCl. Ten times lower detection limit may relate with larger active surface of silver on the surface of CPE, because silver particle probably homogenously saturated CPE. Considering principle of a measurement stripping of AgCl on the surface of CPE modified by silver particle can be expected.

**Figure 4.**
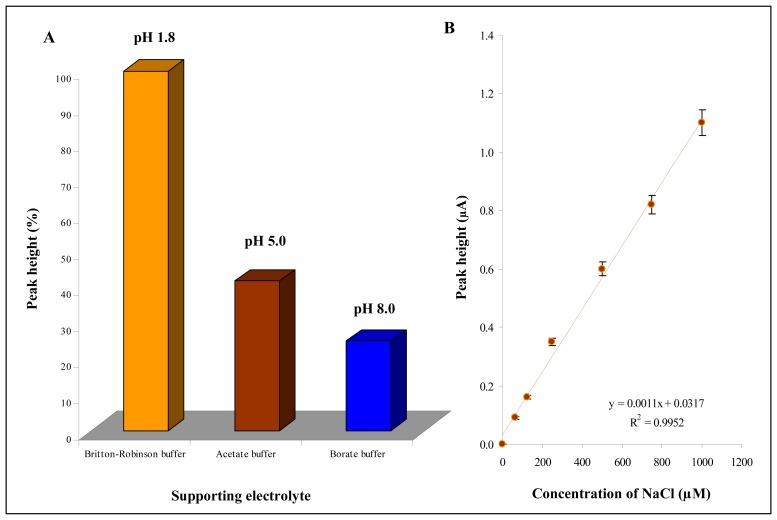
Electrochemical analysis of chloride ions using CPE modified with silver microparticles. (**A**) Effect of type of supporting electrolyte (Britton-Robinson, Acetate and Borate buffer) on chloride ions response. (**B**) Calibration curve.

It can be concluded that CPE modified by silver particles is the most sensitive to the presence of chloride ions. Therefore we optimized basic experimental conditions and used the CPE modified with silver particles. The type of supporting electrolyte was the first condition to be optimized. The highest current response of chloride ions (100 μM) was measured in Britton-Robinson buffer. The height of the chloride peak measured in the presence of acetate and/or borate buffer was for more than 50 % lower compared to Britton-Robinson peak (Figure 4A). The pH of the supporting electrolyte also influenced the chloride ion signal. Hence, optimization of pH of Britton-Robinson buffer was our second step. Higher current responses were determined at lower pH values. Moreover relative standard deviation enhanced with increasing pH of the buffer from 1.2 % to 4.5 % (*n* = 5). Dependence of chloride ions peak height on its concentration measured in Britton-Robinson buffer (pH 1.8) is shown in Figure 4B. The dependence was strictly linear (y = 0.0011x + 0.0317, R^2^ = 0.9952). Due to optimisation steps limit of detection (3 S/N) was lowered and estimated as 1 μM NaCl.

### 3.4 Miniaturization of measuring device

**Figure 5.**
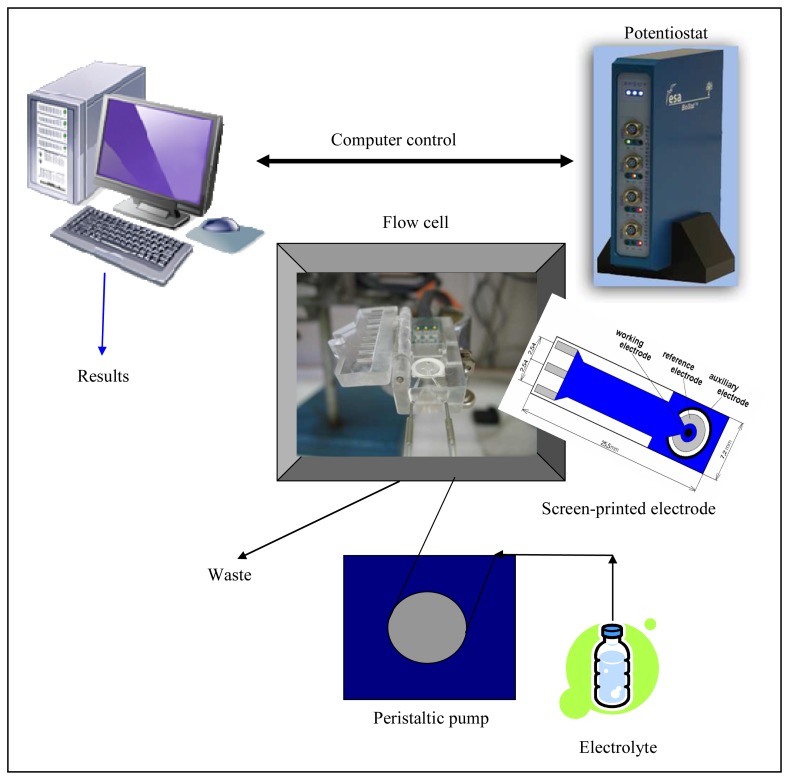
Scheme of the miniaturized flow system.

After we successfully optimized the modifications of CPEs which were connected to a standard laboratory potentiostat, we approached the miniaturization of the whole electrochemical instrument. The measurements were carried out on miniaturized instrument consisted of a Biostat potentiostat with dimensions 35 × 166 × 125 mm, screen printed electrodes, a peristaltic pump and a PC with control software (Figure 5). The electrochemical flow cell was connected to the peristaltic pump via capillary tubing. The pump introduced the supporting electrolyte and samples. Screen printed electrodes with silver electrode as working one were inserted into the cell. The electrodes were connected to miniaturized potentiostat controlled by PC, where electrochemical responses were recorded. To obtain well repeatable responses full immersing of screen printed electrodes was needed.

**Figure 6.**
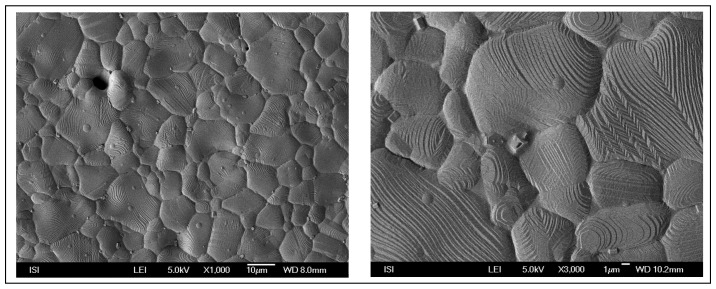
Surface electron microscopic microphotograph of silver working electrode surface (left), in detail (right).

A surface electron microscopic microphotograph of the fabricated Ag-based working electrode surface is shown in Figure 6. Planar screen printed electrodes are intended for one use only, but it is more convenient to utilize them several times for measurements. Therefore we focused on a way to recycle the electrodes. The electrode surface was washed with MiliQ water and polished mechanically with alumina between two independent measurements.

Using this cleaning cycle NaCl (0.5 mM) was detected forty times. The signal decreased with increasing number of measurements. However relative standard deviation was below 11 %. These electrodes can be used for more than tens measurements without considerable lost in height of peak of interest. Cost per one analysis of chlorides in sample is thus markedly reduced. In addition new possibilities of proposing of easy to use and low cost instruments for on-line monitoring of environment are opened.

#### Influence of applied potential and chloride concentrations

**Figure 7.**
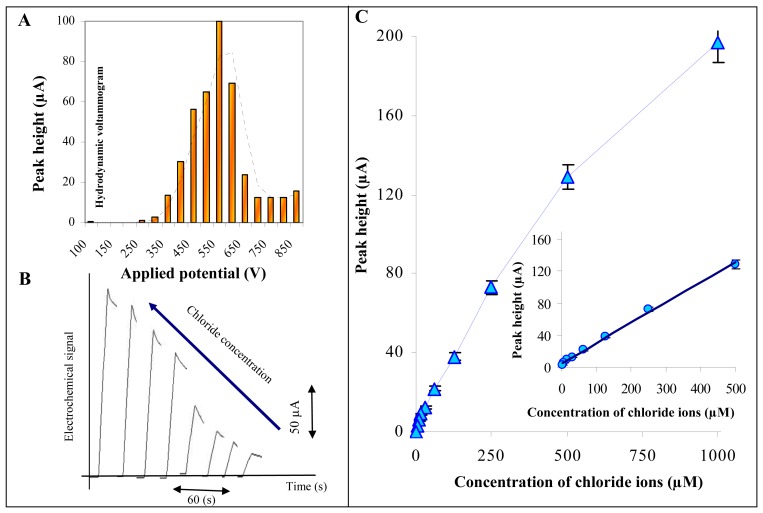
Chloride ion detection using screen printed electrodes. (**A**) Hydrodynamic voltammogram of chloride ions (100 μM). (**B**) Amperometric signals of chloride ions (1000, 500, 250, 125, 63, 32, 15 and 7 μM). (**C**) Calibration curve.

Based on the above mentioned results we choose Britton-Robinson buffer (pH 1.8) as supporting electrolyte. It follows from the voltammograms shown in Figures 2 and 3 that the highest current response was detected within the range of potentials from 150 to 250 mV. Due to the change in the electrode system we investigated on the influence of applied potential (from 100 to 900 mV) on chloride ion peak height. The hydrodynamic voltammogram obtained is shown in Figure 7A. Marked enhancement of chloride ions peak was observed from 350 mV. The highest peaks were measured at 600 mV. Under potentials higher than 650 mV, current response decreased (Figure 7A). It clearly follows from the results obtained that the most suitable potential for detection of chloride ions was 600 mV.

Under the most suitable experimental conditions (Britton-Robinson buffer, pH 1.8 and working electrode potential 600 mV) we studied the effect of different chloride ions concentration on its response. Characteristic amperometric responses are shown in Figure 7B. Before two independent measurements the electrode was polished as mentioned above and, moreover, we had to wait to establish base line. The whole process didn't take more than 5 min. A typical calibration curve is shown in Figure 7C. Concentrations higher than 500 μM form multilayers on the working electrode surface, which results in a slight decrease in peak height. The calibration curve obtained within the range from 5 to 500 μM was strictly linear with the following equation y = 0.2534x + 5.011; R^2^ = 0.9969, relative standard deviation 7.5 %. The limit of detection (3 S/N) was estimated as 500 nM NaCl.

## 4. Conclusions

From the electrodes used for detection of chloride ions screen printed ones connected to miniaturized potentiostat is the most sensitive. This instrument also represents easy-to-use and well portable device for monitoring of chloride ions concentration in various types of samples. Environment is polluted with many toxic compounds contained chloride ions such as persistent organic pollutants (POPs), detergents, and other chemicals.

**Figure 8.**
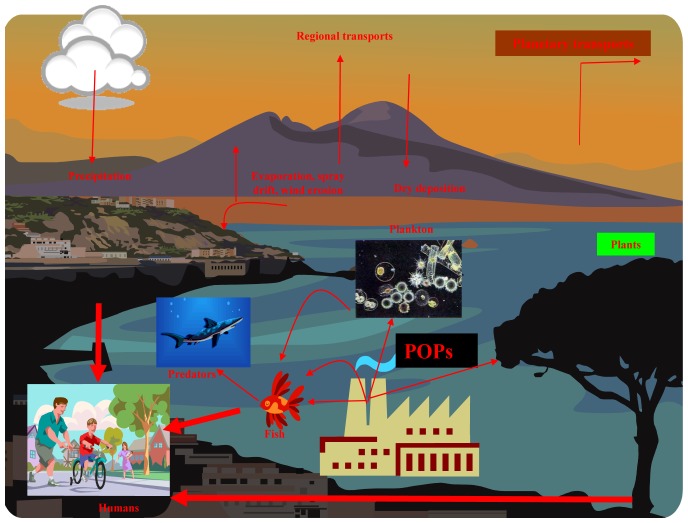
Schematic of the POP cycle in the environment. POPs are released from local sources and transported via atmosphere. They enter locally into water ecosystems (particularly plankton); plankton is subsequently consumed by fish. Predators including humans are also affected because they stand in the top of food chain. The second way is surface contamination of plants.

POPs are chemical substances that persist in the environment, bioaccumulate through the food web, and pose a risk of causing adverse effects to human health and the environment. In light of evidence of long-range transport of these substances to regions where they have never been used or produced, several international communities and organizations have called for urgent global actions to reduce and eliminate releasing of these chemicals. Their cycle in environment is well established. In the latter half of the 20^th^ century these compounds presented considerable technological progress. One of the most “famous” POP molecules is DDT (Dichloro-Diphenyl-Trichloroethane), a general-purpose insecticide toxic to most insect pests. DDT was used with great effect to control mosquitoes responsible for spreading malaria, typhus, and other insect-borne diseases among populations. Nevertheless, after long-lasting exposition it has been demonstrated that POPs may cause may cause cancer and that their agricultural use was a threat to wildlife, particularly birds. Based on these findings usage of POPs has been banned in most of all countries on the world [79-85]. Nowadays, POPs still persist in planetary cycle with continuing entries from various sources (Figure 8).

**Figure 9.**
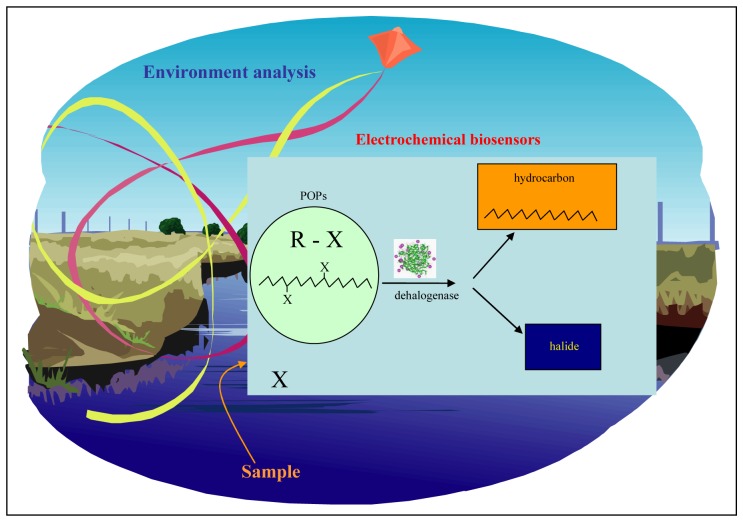
Suggestion of enzyme biosensor for analysis of POPs Pollutant is introduced on the biosensor, where enzymatic cleavage of the pollutant takes place. Hydrocarbon and chloride are released during reaction. Released chloride is detected.

Recently, it was published that enzymatic cleavage of these POPs into the corresponding hydrocarbon and halide can be successfully utilized for indirect detection of these toxic compounds [30]. The enzymes used for this purposes are involved in biochemical pathways enabling bacteria to utilize halogenated compounds via releasing halogen ion from the molecule of halogenated hydrocarbon [86-92]. A scheme of a biosensor using dehalogenase and our miniaturized instrument is shown in Figure 9.
